# Promoting the science of One Health

**DOI:** 10.1038/s41467-023-40293-y

**Published:** 2023-08-07

**Keywords:** Epidemiology

## Abstract

One Health refers to the interconnectedness of the health of humans, animals, and the environment. It recognises that coordination across sectors is needed for effective prevention, detection, and management of infectious disease threats. Although the concept of One Health is not new, it has gained increased prominence following the COVID-19 pandemic, prompting national and international institutions to adopt One Health policies aimed at preventing disease spillover^1–4^. At *Nature Communications*, we have launched a Collection and call for papers in recognition of the need for better scientific evidence to support One Health policy ambitions.

Disease spillover occurs when pathogens, such as influenza and coronaviruses, are transmitted between animals and humans^5,6^. New opportunities for disease spillover can be brought about by environmental changes that result in closer interactions between humans and animals, and a One Health approach is therefore important for prevention and early identification of spillover events^7^. However, there is a need for better scientific evidence to support prevention efforts, by answering questions such as where spillover is most likely to occur, what characteristics of pathogens and animals make them likely to be involved in spillover, and what interventions are effective in prevention of spillover.

A related issue is the emergence of existing infectious diseases in new environments, for example the increasing spread of West Nile virus in Europe^8^. This may happen when new environmental niches for zoonotic disease vectors or animal reservoirs arise due to changes in climate or land use^9^. When transmission events occur close to highly populated or interconnected cities, they are at increased risk of escalating to large-scale outbreaks. To prevent widespread epidemics, we need better information on aspects of disease control such as how to promptly detect new incursions of infectious diseases, understanding what factors promote further spread, and how spread can be restricted.

“At *Nature Communications*, we have launched a Collection and call for papers in recognition of the need for better scientific evidence to support One Health policy ambitions.”

Another focus of One Health-related policies is antimicrobial resistance and use^10^. Antimicrobial resistance is a major threat to human and animal health and can be present in environmental reservoirs, for example in land contaminated with wastewater or where antimicrobials are used in agriculture^11^. Key One Health scientific considerations in this area include monitoring emerging resistance markers through integrated surveillance, understanding mechanisms of transmission of resistance between species, ensuring the safety of the food chain, and developing methods to safely reduce antibiotic use across sectors.

Addressing One Health research questions often requires combining tools and approaches from different areas of science. As the scientific field of One Health research develops, it is also worth considering whether more formal frameworks for these studies could be adopted. For example, are there specific requirements for studies to be classified as One Health research, how can One Health considerations be best built into study designs, and how can evidence from different domains be appropriately triangulated to support actions. As a multidisciplinary journal, *Nature Communications* welcomes submission of articles across the spectrum of One Health science. We also encourage submissions aiming to develop best practices in the conduct and reporting of these studies.
